# Changes of trace element status during aging: results of the EPIC-Potsdam cohort study

**DOI:** 10.1007/s00394-019-02143-w

**Published:** 2019-11-30

**Authors:** Julia Baudry, Johannes F. Kopp, Heiner Boeing, Anna P. Kipp, Tanja Schwerdtle, Matthias B. Schulze

**Affiliations:** 1 Department of Molecular Epidemiology, German Institute of Human Nutrition Potsdam-Rehbruecke, 14558 Nuthetal, Germany; 2 TraceAge-DFG Research Unit on Interactions of Essential Trace Elements in Healthy and Diseased Elderly, Potsdam-Berlin-Jena, Germany; 3 Department of Food Chemistry, Institute of Nutritional Science, University of Potsdam, 14558 Nuthetal, Germany; 4 Department of Epidemiology, German Institute of Human Nutrition Potsdam-Rehbruecke, 14558 Nuthetal, Germany; 5 Department of Molecular Nutritional Physiology, Institute of Nutritional Sciences, Friedrich Schiller University Jena, 07743 Jena, Germany

**Keywords:** Aging, Copper, Determinants, Iodine, Iron, Manganese, Selenium, Trace element profiles, Zinc

## Abstract

**Purpose:**

We aimed to evaluate age-dependent changes of six trace elements (TE) [manganese (Mn), iron (Fe), zinc (Zn), copper (Cu), iodine (I), and selenium (Se)] over a 20-year period.

**Methods:**

TE concentrations were determined using repeated serum samples taken at baseline and after 20 years of follow-up from 219 healthy participants of the EPIC-Potsdam study, using inductively coupled plasma tandem mass spectrometry. For each TE, absolute and relative differences were calculated between the two time points, as well as the proportion of individuals within normal reference ranges. Interdependence between age-related TE differences was investigated using principal component analysis (PCA). Relationships between selected factors (lifestyle, sociodemographic, anthropometric factors, and hypertension) and corresponding TE longitudinal variability were examined using multivariable linear regression models.

**Results:**

Median age of our study sample was 58.32 years (4.42) at baseline and 40% were females. Median Mn, Zn, Se concentrations and Se to Cu ratio significantly decreased during aging while median Fe, Cu, I concentrations and Cu to Zn ratio significantly increased. A substantial percentage of the participants, at both time points, had Zn concentrations below the reference range. The first PCA-extracted factor reflected the correlated decline in both Mn and Zn over time while the second factor reflected the observed (on average) increase in both Cu and I over time. Overall, none of the investigated factors were strong determinants of TE longitudinal variability, except possibly dietary supplement use, and alcohol use for Fe.

**Conclusions:**

In conclusion, in this population-based study of healthy elderly, decrease in Mn, Zn, and Se concentrations and increase in Fe, Cu, and I concentrations were observed over 20 years of follow-up. Further research is required to investigate dietary determinants and markers of TE status as well as the relationships between TE profiles and the risk of age-related diseases.

**Electronic supplementary material:**

The online version of this article (10.1007/s00394-019-02143-w) contains supplementary material, which is available to authorized users.

## Introduction

Essential trace elements (TE) are micronutrients that are found in very small amounts in the human body. They play indispensable roles for maintaining human health as they are involved in various metabolic processes and signaling pathways [1]. Foods and beverages, which are the main suppliers of TE, contain a combination of diverse TE. It is also clearly established that TE interact with each other. For instance, interventional studies have shown that iron (Fe) supplementation along with iodine (I) supplementation markedly mitigates thyroid-related disorders [2]. Another example is the high competition between copper (Cu) and zinc (Zn); Zn ingestion has thus been shown to reduce Cu absorption via the modulation of the expression of metallothioneins [3].

In turn, the inappropriate supply of TE (deficiencies or excess) has been associated with several health disorders [4, 5]. A large number of factors influence TE homeostasis including dietary intake, sex, health status or age [6]. Alternatively, several TE can affect the process of aging [6] by contributing to numerous age-related mechanisms, via, for example, their role in the immune system or against oxidative stress. They have been, therefore, hypothesized to contribute to the pathogenesis of age-related chronic diseases [6–11].

TE status can be, to some extent, modulated by intake. Given that older individuals have specific nutrient requirements and that their proportion will reach around one-sixth of world population by 2050 [12], studying age-dependent changes of TE profiles is of great importance. Indeed, understanding how TE status relates to aging will help to improve the TE status of the elderly and will help to better prevent age-related decline.

With regard to age-related differences, consistent evidence has been observed on specific TE, selenium (Se) concentrations appear to be lower in older adults in comparison to younger adults in some studies [13, 14], while varying results have been observed across sexes in a French study [15]. Lower Zn concentrations among older persons have been observed [16, 17], presumably due to age-related epigenetic changes in Zn transporter genes [18]. High plasma Cu associated with low plasma Zn concentrations [17, 19–22] have also been consistently reported in the elderly, leading some authors to consider Cu to Zn ratio as a suitable biomarker for aging [21]. In the representative US National Health and Nutrition Examination Survey, higher blood manganese (Mn) concentrations were significantly associated with younger age [23]. Data regarding age-related differences for I are scarce, while Fe deficiency anemia or Fe overload is frequent in older age [24].

Interestingly, most of the studies on age and TE profiles have been carried-out cross-sectionally. One of the few longitudinal studies on determinants of age-dependent changes in TE was conducted in French adults over a period of 9 years, based on plasma Se. The decrease in plasma Se over time was associated with occurrence of cardiovascular diseases, but not with sociodemographic and lifestyle characteristics, diabetes, hypertension or dyslipidemia. On top of that, very few studies have considered several TE simultaneously.

Therefore, from these figures, it is not clear whether aging is accompanied by a specific shift of TE profiles and what is the structure of these changes as well as their magnitude.

Hence, we aim to examine age-dependent changes of TE profiles, over a 20-year period, in healthy men and women. We focus on six essential and health relevant TE, namely Mn, Fe, Zn, Cu, I, and Se. We first characterize absolute and relative TE differences over time and investigate interdependence of TE changes within the TE profile. We further examine sociodemographic, lifestyle and health factors associated with TE longitudinal variability.

## Subjects and methods

### Study population and design

We used longitudinal data from the European Prospective Investigation into Cancer and Nutrition (EPIC)-Potsdam study. The objectives and the methodology of the EPIC-Potsdam study have been detailed elsewhere [25]. In brief, the EPIC-Potsdam study is part of the prospective ongoing multicenter EPIC study which aims to investigate the relationships between nutrition, cancer, and other chronic diseases. The EPIC-Potsdam study comprises 27,548 subjects (16,644 women and 10,904 men) from the general population in Potsdam and surroundings. At recruitment (1994–1998), blood samples, anthropometric measurements, as well as sociodemographic, lifestyle, and dietary and health information were collected from the study participants [25].

Beginning in 2014, participants from the cohort were re-examined. This re-examination included repeated anthropometric measurements, blood sample collection as well as collection of data on health and medication status. Among all participants who were re-examined until March 2017 with available blood samples at baseline and after re-invitation (*n* = 2418), 220 healthy individuals were randomly selected (aged 35–65 year at baseline). Further inclusion criteria included no-active smoking as well as no-use of specific medication (antibiotics, metformin, and statins) at the two time points.

### Assessment of sociodemographic and lifestyle data

At enrolment, sociodemographic, lifestyle data (sex, age, smoking status, alcohol consumption, occupation, education level and physical activity) were collected using self-administered questionnaires. Prevalent hypertension was assessed using blood pressure and self-report medication. Anthropometric data (height, weight, waist and hip circumferences) were assessed by trained staff. Same information was collected at 20-year re-examination. Occupational status was not inquired in the context of the re-examination, and data from the 5th follow-up questionnaire were used.

### Assessment of dietary data

At baseline, a diet questionnaire (food frequency questionnaire, FFQ) measuring usual diet over the past 12 months was administered to the participants to collect information on amount and frequency of food and beverage intake [26, 27]. Information regarding supplement use was also collected from this FFQ. In particular, participants were asked whether they were regularly (continuously for at least 4 weeks) taking the following preparations: mineral tablets (yes/no), vitamin tablets (yes/no).

A diet score (referring to the Mediterranean diet score adapted to non-Mediterranean populations) was computed at baseline to study overall healthy diet as a determinant of TE status. Construction of this score has been fully described elsewhere [28]. Briefly, the Mediterranean diet score (range 0–18 points) consisted of nine food components (namely vegetables, cereals, fruits and nuts, fish, legumes, meat, dairy products, alcohol, and olive oil), corresponding to sex-specific tertiles of intakes. For beneficial food components (fruits and nuts, vegetables, legumes, fish, and cereals), 0, 1 or 2 points were assigned for belonging to the first, second, and third tertile of intake, respectively, and conversely for meat and dairy products. With regard to alcohol consumption, moderate intake was considered as ideal, and 0 point was attributed to non-olive oil consumers, and 1 and 2 for those with intake below or above the median.

Furthermore, 1 year after re-examination (2014–2016), participants were asked, through a questionnaire, to report their medication and supplement use in the past 12 months. They were specifically asked to answer the following question: “Have you taken any vitamin supplements, mineral supplements or other food supplements in the past 12 months over a period of at least one month?”. If so, participants had to tick among different compounds, including multivitamin preparation and mineral preparation.

### Measurement of TE profiles

TE profiles were assessed using repeated serum samples taken at baseline and after re-invitation from participants.

#### Method of TE profiling

All the samples (baseline and follow-up) were blinded and mixed in a single large set and were frozen at − 80 °C until 1 day prior to measurement. Same instruments, pipettes, operators, vessels, and chemicals were used for all the samples. Samples were kept at 4 °C until the next day for measurement. For TE-profiling, the method published in the *Journal of Trace Elements in Medicine and Biology* was employed [29]. In brief, 50 µL of sample was diluted with 440 µl of a diluent solution containing 5 vol.% 1-butanol (99%, Alfa Aesar, Karlsruhe, Germany), 0.05 m % Na-EDTA (Titriplex^®^ III, pro analysis, Merck, Darmstadt, Germany), 0.05 vol.% Triton™ X-100 (10% in H_2_O, Merck-Sigma Aldrich, Steinheim, Germany), and 0.25 vol% ammonium hydroxide (puriss. p.a. plus, 25% in H_2_O, Fluka, Buchs, Germany). As internal standard and for isotope dilution analysis 10 µL of a solution containing 50 µg/L ^77^Se (prepared from isotopically enriched ^77^Se standard: 97.20 ± 0.20% ^77^Se; 0.10% ^74^Se; 0.40 ± 0.10% ^76^Se; 2.40 ± 0.10% ^78^Se; 0.10% ^80^Se; 0.10% ^82^Se as certified by Trace Sciences International, Ontario, Canada, purchased from Eurisotop SAS, Saarbrücken, Germany) and 5 µg/L Rh (diluted from 1000 mg/L single element stock solution, Carl Roth, Karlsruhe, Germany) was added to give a total volume of 500 µL. This solution was directly subjected to analysis via inductively coupled plasma tandem mass spectrometry (ICP-MS/MS) (Agilent ICP-QQQ-MS 8800, Agilent Technologies, Waldbronn, Germany). ICP parameters and monitored isotopes can be found in Supplemental Table 1. The instrument was optimized daily for maximum sensitivity. For external calibration (all elements except Se), standards were prepared matrix-matched in the diluent solution using 1000 mg/L single element stock solutions, purchased from Carl Roth (Karlsruhe, Germany). Se was determined using isotope dilution analysis. For quality control, reference material RECIPE^®^ ClinChek^®^ serum control lyophilized (Ref. 8880-8882, Lot 347) (both levels) was measured in triplicate daily. Mean recoveries were Mn: 106.0% ± 8.9%, Fe: 105.4% ± 6.4%, Cu: 101.1% ± 6.4%, Zn: 91.6% ± 8.4%, I: 92.0% ± 9.0%, Se: 76.5% ± 20.3%. Furthermore, sufficient blank samples (distilled H_2_O) were carried along to determine limits of detection (LOD, 3ϭ-criterion) and quantification (LOQ, 10ϭ-criterion) on a daily basis. Measurement was done in seven single-day batches with 70 samples each, except for the last one, which only had 54 left.

### Ethics

Consent was obtained from all participants of the study, and approval was given by the Ethical Committee of the State of Brandenburg, Germany.

### Statistical analysis

#### Selection of the analytical study sample

Across the 220 selected participants, one individual with a concentration of I at the second time point ten times higher than the P99 was excluded from the analysis. The final sample included 131 men and 88 women.

#### Treatment of left-censored and missing data

For Mn, 8 concentration values were below the limit of detection (LOD) and 19 below the limit of quantification (LOQ) at baseline, and 5 concentration values were below the LOD and 36 below the LOQ at the second time point. Left-censored data were handled by substituting by LOD/√2 for censored values less than LOD and by LOQ/√2 for censored values less than LOQ. In addition, for all TE, except Se, since it was determined by isotope dilution analysis, data were also missing due to insufficient recovery of the internal standard, indicating strong matrix effects in these samples, thus rendering the obtained values unreliable (10 missing at baseline and 8 at 20 years of follow-up). Missing values were handled using single imputation based on the fully conditional specification method [30].

#### Descriptive statistics

Descriptive characteristics were presented as mean [standard deviation (SD)] for parametric continuous variables, median [interquartile range (IQR)] for non-parametric continuous variables and percentages for categorical variables. The normality of the variables was assessed using the Kolmogorov–Smirnov test, histograms, Q–Q plots, and box-plots. Whenever available, characteristics over time were compared using paired *t* test, non-parametric Wilcoxon signed-rank test, or using the matched pair McNemar’s test, as appropriate.

#### Age-dependent changes in TE profiles

Baseline, follow-up concentrations and differences for each TE (ΔTE, defined as the differences between the follow-up concentration and the baseline concentration, at the individual level) were analyzed as continuous variables. As most TE variables at the two time points showed non-normality, concentrations were presented as median (IQR), and differences in TE profiles over time were evaluated using Wilcoxon signed-rank test. In addition, medians of the relative differences (IQR), expressed as %, were computed to estimate the effect size of the differences. Serum Cu to Zn ratio as well as serum Cu to Se ratio was calculated as they may constitute novel biomarkers for aging [21] and resistance to thyroid hormone β [31], respectively.

We also calculated the proportion of subjects (in %) with concentrations within normal reference ranges, at the two time points [32–37]. Of note, reference ranges for Mn vary among laboratories [38]. We further calculated the percentage of participants at both time points with optimal Se status (defined as Se concentrations > 100 µg/L, based on both saturation of glutathione peroxidase activity and selenoprotein P [39]), as well as the percentage of participants with Cu to Zn ratio ≤ 2 (a ratio > 2 expressing inflammatory reaction or inadequate Zn status [40]). The percentages at baseline and follow-up were compared using the matched pair McNemar’s test. *P* values were adjusted for multiple testing using the Benjamini–Hochberg procedure.

#### Characterization of interdependence of age-related changes in TE profiles

Spearman’s correlations were undertaken for investigating the relationships between changes in individual TE. Associations within differences in TE were further examined using Spearman-adjusted partial correlation coefficients, adjusted for all other TE differences.

Principal component analysis (PCA) was performed on the age-related differences in each individual TE (*i.e.* 6 variables constituting the absolute differences in concentration of each TE between the two time points), using the PROC FACTOR^®^ procedure in SAS. This method allows to generate independent linear combinations of the initial variables (here the age-related differences in TE concentrations), thereby maximizing the explained variance. The factors were orthogonally rotated using the ‘VARIMAX option’ in SAS. The number of factors retained was based on eigenvalue > 1, a scree-test, and the interpretability of factors.

#### Factors associated with longitudinal change in TE status

For comparability, TE differences as well as continuous independent variables were standardized. Assumptions of linear regressions were checked using diagnostic plots (fit plot, residual plot, and a diagnostics panel from the PROC REG^®^ procedure in SAS). They were not verified for Mn, I and Se, Se to Cu ratio and, therefore, confidence intervals for the parameters for these TE differences need to be taken cautiously. Multivariable linear regression models were then used to analyze sociodemographic, lifestyle and anthropometric characteristics associated with longitudinal TE variability.

The following baseline factors were included into the models: concentration of the respective TE, sex, educational level (no vocational training/vocational training, technical college, university), age, waist circumference, overall leisure-time physical activity (defined as the sum of sports, biking, and gardening in h/week), and dietary quality (as assessed by the Mediterranean diet score). In addition, for variables available at both baseline and follow-up, change over time was also considered, *i.e.* change in mineral and vitamin use (non-use/use at baseline/use at follow-up/constant use), change in hypertension status (non-hypertensive/hypertensive/newly hypertensive), change in alcohol consumption (no or low intake/constant high intake/stop drinking/higher intake at follow-up/lower intake at follow-up), change in amount of time devoted to sports, and change in waist to hip ratio during aging. For all statistical analyses, a *P* value < 0.05 was considered statistically significant. All statistical analyses were performed using the statistical software package SAS (version 9.4, Enterprise Guide 6.1, SAS Institute Inc., Cary, NC, USA), with a significance level of 0.05 for 2-sided tests.

#### Sensitivity analyses

To evaluate to what extent the single imputations have affected the results, we analyzed the age-dependent changes in TE status and characterized the independence of TE changes excluding participants with missing concentrations related to insufficient recovery of the internal standard (final sample, *N* = 203) (Supplemental Tables 2–4, Supplemental Fig. 1).

## Results

### Characteristics of the participants

The median follow-up time (IQR) of our study sample was 18.9 years (1.98). Characteristics of the participants at baseline and after ~ 20 years of follow-up are shown in Table 1. The median (IQR) age of our study sample was 58.32 years (4.42) at baseline and 77.60 years (3.93) at the second time point, and 40% of the participants were females. Height of the participants significantly decreased over time, whereas waist circumference and waist to hip ratio significantly increased. No significant change over time was observed for body weight and hip circumference. The percentage of participants with history of hypertension significantly increased during aging. Most of the participants at baseline had a full-time job (50%) and were retired at 20 years of follow-up (91%). Most of them were holders of a university degree (42%) and reported low consumption of alcohol at both time points. Median baseline leisure physical activity was relatively high (6 h per week). The proportion of vitamin users was stable (20–25%) while the proportion of mineral users significantly increased over time. The median (IQR) of the Mediterranean diet score of the sample at baseline was 9 (4).

**Table 1:** Descriptive characteristics of the sample at baseline and at 20 years of follow-up, EPIC-Potsdam, imputed sample, *N* = 219

	At baseline (1994–1998)	At 20 year follow-up (2014–2016)	*P* value^a^
Female (%)	40.18	40.18	
Age (years)*	58.32 (4.42)	77.60 (3.93)	
Height (cm)¶	168.93 (8.47)	166.94 (8.74)	< 0.0001
Body weight (kg)¶	76.33 (11.53)	75.64 (12.56)	0.15
Waist to hip ratio*	0.90 (0.17)	0.95 (0.13)	< 0.0001
Waist circumference (cm)¶	89.99 (11.15)	95.47 (11.89)	< 0.0001
Hip circumference (cm)*	100.50 (9.00)	99.00 (9.00)	0.08
BMI (kg/m^2^)¶	26.72 (3.50)	27.13 (4.06)	0.005
Hypertension, yes (%)	51.14	62.10	< 0.0001
Occupation (%)			< 0.0001
Full time (≥ 35 h/week)	50.23	1.37	
Part time (15– < 35 h/week)	3.65	1.37	
Hourly (< 15 h/week)	4.11	0.46	
Jobless/retraining	10.96	0	
(Early) retirement/invalidity pension	30.59	91.32	
Unemployed	0.46	5.48	
Education level (%)			
University degree	42.01	–	
Trade/technical school	28.77	–	
No degree/vocational training	29.22	–	
Leisure-time physical activity (h/week)*	6.00 (9.00)	–	
Use of vitamin supplement, yes (%)	20.55	25.11	0.17
Use of mineral preparation, yes (%)	13.24	29.22	< 0.0001
Mediterranean diet score (max = 18 points)*	9.00 (4.00)	–	
Alcohol consumption (%)			0.02
Abstainers	0.46	4.11	
Low consumption (≤ 12 g for women, ≤ 24 g for men)	78.08	79	
High consumption (> 12 g for women, > 24 g for men)	21.46	16.89	

Data are medians (IQR)* or means (SD)¶ for continuous variables or % for categorical variables, as appropriate. Data were missing at follow-up (*n*_missing_ = 1 for occupation; *n*_missing_ = 5 for mineral and vitamin use; *n*_missing_ = 8 for alcohol consumption; < 4% for all variables) and were imputed using a single imputation procedure (fcs procedure)

– Data were not available at the second time point

^a^*P* values based on paired *t* test or non-parametric Wilcoxon signed-rank test, as appropriate

### Age-dependent change in TE profile

Median TE concentrations at baseline and at 20 years of follow-up are presented in Table 2. Median Mn, Zn, Se concentrations and Se to Cu ratio significantly decreased during aging (all *P* values < 0.002) while median Fe, Cu, I concentrations and Cu to Zn ratio significantly increased over time (all *P* values < 0.003). Changes were most pronounced for Fe (median increase by 17%) and Mn (median decrease by 16%).

**Table 2:** Serum concentrations of measured TE at baseline and at 20 years of follow-up, EPIC-Potsdam, imputed sample, *N* = 219

	At baseline (1994–1998)	At 20 year follow-up (2014–2016)	*P* value^a^	Absolute difference^b^	Relative difference^c^ (%)
Manganese (µg/L)	1.08 (1.11)	0.83 (0.92)	0.0016	− 0.14 (1.19)	− 16 (111)
Iron (µg/L)	965 (446)	1114 (372)	< 0.0001	161 (472)	17 (55)
Copper (µg/L)	989 (226)	1039 (240)	< 0.0001	54.0 (188.0)	5 (20)
Zinc (µg/L)	704 (175)	636 (154)	< 0.0001	− 48.0 (199)	− 8 (26)
Iodine (µg/L)	47.68 (11.69)	49.24 (11.07)	0.0026	1.90 (9.31)	5 (20)
Selenium (µg/L)	85.19 (17.15)	79.28 (17.69)	< 0.0001	− 5.61 (16.1)	− 7 (18)
Cu to Zn ratio	1.43 (0.48)	1.62 (0.53)	< 0.0001	0.21 (0.49)	14 (37)
Se to Cu ratio	0.09 (0.02)	0.08 (0.02)	< 0.0001	− 0.01 (0.02)	− 10 (24)

Data are medians (IQR)

*Cu to Zn ratio* Copper to Zinc ratio, *Se to Cu ratio* Selenium to Copper ratio, *TE* Trace element

^a^*P* values based on non-parametric Wilcoxon signed-rank test

^b^Differences between the follow-up concentration and the baseline concentration

^c^Differences between the follow-up concentration and the baseline concentration, with respect to the baseline concentration, multiplied by 100

### Percentages of participants with TE concentrations within reference ranges

The proportion of individuals within the reference range for Mn was significantly higher at the second time point while these percentages were similar over time for Fe, Cu, and I (all % > 80) (Table 3). A substantial percentage of the participants at baseline and at 20 years of follow-up had Zn concentrations outside the reference range (~ 40% below the normal range at baseline and more than 50% after 20 years, data not shown). The great majority of participants had Cu to Zn ratio ≤ 2, and this percentage significantly decreased over time. With regard to Se, the percentages of individuals within the reference range significantly decreased during aging (90% at baseline *vs.* 75% after 20 years of follow-up). Only 12.79% of the participants at baseline and 6.39% at follow-up had concentrations of Se considered as optimal.

**Table 3:** Percentage of individuals within the reference ranges at baseline and at 20 years of follow-up, EPIC-Potsdam, imputed sample, *N* = 219

	Normal reference range [µg/L]	% at baseline	% at 20 year follow-up	Crude *P* value^a^	Adjusted *P* value^b^
Manganese	0.15–2.65 [32]	85.84	93.15	0.0077	0.0205
Iron	Men: 550–1600; Women: 400–1550 [37]	91.32	92.24	0.7316	0.7316
Copper	637–1401.2 [36]	91.78	94.98	0.0896	0.1195
Zinc	660–1100 [35]	52.97	42.47	0.0195	0.0312
Iodine	40–92 [34]	84.47	88.13	0.1944	0.2222
Selenium	70–150 [33]	89.95	75.34	< 0.0001	0.0002
Optimal status	> 100 [39]	12.79	6.39	0.0164	0.0312
Cu to Zn ratio	≤ 2 [40]	91.78	83.11	0.0018	0.0072

*Cu to Zn ratio* Copper to Zinc ratio

^a^*P* values based on McNemar’s test

^b^False Discovery Rate adjusted *P* values

### Interdependence of age-related change in serum TE-concentrations

Spearman’s correlation coefficients between age-related differences in serum TE-concentrations are shown in Table 4. Correlations between age-related differences in TE were low to modest and ranged from − 0.01 to 0.30. The strongest correlation was observed for ΔI with ΔCu. Adjusted Spearman’s partial correlations yielded similar trends (Supplemental Table 5).

**Table 4:** Spearman correlations between TE differences over time, EPIC-Potsdam, imputed sample, *N* = 219

		ΔMn	ΔFe	ΔCu	ΔZn	ΔI	ΔSe
ΔMn	r	1					
	P	.					
ΔFe	r	0.02	1				
	P	0.75	.				
ΔCu	r	0.09	− 0.01	1			
	P	0.17	0.87	.			
ΔZn	r	**0.12**	**0.12**	**0.12**	1		
	P	0.07	0.07	0.09	.		
ΔI	r	− 0.01	0.01	**0.30**	**0.20**	1	
	P	0.85	0.91	< 0.0001	0.003	.	
ΔSe	r	0.00	0.00	0.04	**0.12**	0.07	1
	P	0.96	0.96	0.54	0.08	0.28	.

ΔTE, defined as the differences between the follow-up concentration and the baseline concentration. Correlation coefficients r ≥ 0.10 or r ≤ − 0.10 are displayed in bold

*Cu* Copper, *Fe* Iron, *I* Iodine, *Mn* Manganese, *P* significance or probability value, *r* correlation coefficient, *Se* Selenium, *TE* Trace element, *Zn* Zinc

### Characterization of patterns of TE changes

**Fig. 1:**
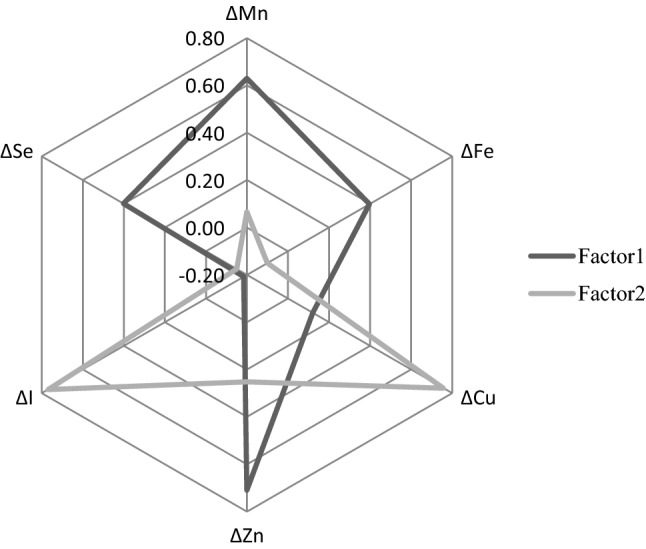
Factor loadings after varimax rotation of the two PCA-derived factors based on TE concentration differences over time, EPIC-Potsdam, imputed sample, *N* = 219. ΔTE, defined as the differences between the follow-up concentration and the baseline concentration. *Cu* Copper, *Fe* Iron, *Mn* Manganese, *I* Iodine, *PCA* Principal component analysis, *Se* Selenium, *TE* Trace element, *Zn* Zinc

Two major TE patterns were identified by PCA that accounted for 42% of the total initial variance of the calculated TE differences over time (Fig. 1). The first factor identified by PCA reflected the correlated decline in both Mn and Zn over time while the second factor reflected the observed (on average) increase in both Cu and I over time.

### Association between sociodemographic, lifestyle and anthropometric factors and longitudinal serum TE change

The associations between factors and longitudinal serum TE change are presented in Table 5. Not surprisingly, respective baseline concentrations were significantly related to longitudinal TE variability. Women had higher increase in Fe and lower increase in Cu over time than men. Vitamin users at the second time point had lower Cu increase while constant users had slightly higher I increase in comparison with non-users. Mineral users at follow-up had lower Fe increase and higher Cu increase, and higher Se decrease than non-users. Mineral users at both time points had higher Se decrease than non-users. Hypertensive individuals had lower decline in Se than non-hypertensive. Compared to low alcohol consumers, heavy consumers at baseline, and at both time points had higher Fe increase. Higher dietary quality (as expressed by higher Mediterranean score), and to a lower extent, higher age at baseline were associated with higher Mn decrease. None of the other studied factors were significantly associated with TE variability.

**Table 5:** Factors associated with changes in TE status, EPIC-Potsdam, imputed sample, *N* = 219

Baseline concentration^a^		Sex (ref = male)	Education (ref = no vocational training/vocational training)		Change in vitamin use (ref = non-use)			Change in mineral use (ref = non-use)			Change in hypertension status (ref = non-hypertensive)	
			Technical college	University	Use at baseline	Use at follow-up	Constant use	Use at baseline	Use at follow-up	Constant use	Hypertensive	Newly hypertensive
ΔMn **− 0.85 (− 0.92; − 0.77)**		0.09 (− 0.12; 0.30)	− 0.06 (− 0.26; 0.15)	0.03 (− 0.16; 0.22)	− 0.12 (− 0.41; 0.17)	− 0.01 (− 0.27; 0.24)	0.13 (− 0.17; 0.43)	0.27 (− 0.06; 0.61)	− 0.05 (− 0.27; 0.17)	− 0.13 (− 0.47; 0.22)	0.06 (− 0.11; 0.23)	0.05 (− 0.22; 0.32)
ΔFe **− 0.67 (− 0.77; − 0.56)**		**0.33 (0.05; 0.6)**	0.09 (− 0.18; 0.36)	0.09 (− 0.16; 0.34)	0.04 (− 0.34; 0.43)	0.2 (− 0.14; 0.54)	0.26 (− 0.14; 0.66)	− 0.35 (− 0.8; 0.1)	**− 0.35 (− 0.64; − 0.06)**	0.1 (− 0.36; 0.56)	0.05 (− 0.18; 0.27)	0.09 (− 0.27; 0.44)
ΔCu **− 0.69 (− 0.81; − 0.56)**		**− 0.47 (− 0.79; − 0.16)**	− 0.05 (− 0.35; 0.24)	− 0.1 (− 0.37; 0.17)	0.03 (− 0.38; 0.43)	**− 0.51 (− 0.87; − 0.14)**	− 0.01 (− 0.43; 0.42)	0.25 (− 0.22; 0.73)	**0.35 (0.05; 0.66)**	0.33 (− 0.15; 0.82)	0.08 (− 0.16; 0.32)	0.17 (− 0.21; 0.55)
ΔZn **− 0.81 (− 0.89; − 0.72)**		0.12 (− 0.09; 0.34)	0.04 (− 0.18; 0.25)	− 0.03 (− 0.23; 0.17)	− 0.04 (− 0.34; 0.26)	0.18 (− 0.09; 0.45)	0.09 (− 0.23; 0.41)	0.35 (0; 0.71)	0.04 (− 0.19; 0.26)	− 0.08 (− 0.45; 0.28)	− 0.03 (− 0.21; 0.15)	0.07 (− 0.21; 0.35)
ΔI **− 0.41 (− 0.54; − 0.28)**		− 0.01 (− 0.35; 0.33)	0.2 (− 0.14; 0.54)	− 0.05 (− 0.36; 0.27)	0.16 (− 0.32; 0.63)	− 0.03 (− 0.46; 0.4)	**0.5 (0.004; 1)**	0.1 (− 0.46; 0.65)	0.04 (− 0.32; 0.4)	− 0.31 (− 0.88; 0.27)	0.04 (− 0.24; 0.33)	− 0.04 (− 0.48; 0.41)
ΔSe **− 0.59 (− 0.71; − 0.48)**		0.01 (− 0.29; 0.3)	0.25 (− 0.05; 0.55)	0.17 (− 0.1; 0.45)	− 0.39 (− 0.81; 0.03)	− 0.36 (− 0.73; 0.02)	− 0.4 (− 0.84; 0.03)	0.21 (− 0.28; 0.7)	**0.47 (0.15; 0.78)**	**0.86 (0.35; 1.38)**	**− 0.28 (− 0.53; − 0.04)**	− 0.18 (− 0.56; 0.21)
Δ(Cu to Zn ratio) **− 0.58 (− 0.7; − 0.46)**		**− 0.53 (− 0.86; − 0.21)**	− 0.11 (− 0.41; 0.2)	− 0.05 (− 0.33; 0.24)	− 0.03 (− 0.46; 0.4)	**− 0.58 (− 0.96; − 0.2)**	− 0.07 (− 0.52; 0.37)	− 0.22 (− 0.72; 0.28)	0.24 (− 0.08; 0.56)	0.43 (− 0.09; 0.94)	0.12 (− 0.13; 0.37)	0.02 (− 0.38; 0.42)
Δ(Se to Cu ratio) **− 0.45 (− 0.59; − 0.32)**		0.2 (− 0.16; 0.56)	0.19 (− 0.15; 0.53)	0.23 (− 0.08; 0.54)	− 0.48 (− 0.95; − 0.02)	**− 0.15 (− 0.57; 0.27)**	− 0.4 (− 0.89; 0.09)	0.13 (− 0.42; 0.67)	0.26 (− 0.09; 0.61)	**0.77 (0.2; 1.35)**	**− 0.29 (− 0.56; − 0.01)**	− 0.32 (− 0.75; 0.12)

	Change in alcohol intake (ref = no or low intake)				Age^a^	Baseline waist circumference^a^	Baseline leisure-time physical activity^a^	Change in amount of time devoted to sport^a^	Change in waist to hip ratio^a^	Baseline mediterranean score^a^
	High intake	Stop drinking	Higher intake at follow-up	Lower intake at follow-up						
ΔMn	− 0.16 (− 0.44; 0.12)	− 0.37 (− 0.78; 0.04)	0.2 (− 0.07; 0.47)	0.1 (− 0.14; 0.34)	**0.08 (0; 0.16)**	0 (− 0.1; 0.1)	0.06 (− 0.08; 0.21)	0.03 (− 0.11; 0.18)	0.04 (− 0.04; 0.12)	**− 0.08 (− 0.16; − 0.01)**
ΔFe	**0.4 (0.03; 0.78)**	− 0.31 (− 0.86; 0.23)	**0.47 (0.11; 0.84)**	0.09 (− 0.23; 0.4)	0 (− 0.11; 0.1)	− 0.06 (− 0.19; 0.07)	− 0.17 (− 0.36; 0.03)	− 0.05 (− 0.25; 0.14)	0.06 (− 0.05; 0.16)	− 0.02 (− 0.12; 0.08)
ΔCu	− 0.12 (− 0.52; 0.29)	− 0.09 (− 0.67; 0.49)	− 0.13 (− 0.52; 0.26)	− 0.08 (− 0.42; 0.26)	0.05 (− 0.06; 0.17)	− 0.07 (− 0.21; 0.07)	− 0.06 (− 0.27; 0.14)	− 0.03 (− 0.23; 0.18)	0.03 (− 0.09; 0.14)	0.01 (− 0.1; 0.12)
ΔZn	0.01 (− 0.28; 0.31)	− 0.4 (− 0.83; 0.03)	− 0.17 (− 0.47; 0.12)	0.05 (− 0.2; 0.3)	− 0.07 (− 0.15; 0.02)	− 0.01 (− 0.11; 0.1)	− 0.11 (− 0.27; 0.04)	− 0.07 (− 0.22; 0.08)	− 0.02 (− 0.11; 0.06)	− 0.02 (− 0.1; 0.06)
ΔI	− 0.04 (− 0.5; 0.43)	0.38 (− 0.31; 1.06)	− 0.29 (− 0.74; 0.17)	− 0.22 (− 0.62; 0.17)	0 (− 0.14; 0.13)	0.05 (− 0.11; 0.22)	− 0.22 (− 0.46; 0.02)	− 0.17 (− 0.41; 0.07)	− 0.08 (− 0.21; 0.06)	0.03 (− 0.09; 0.16)
ΔSe	− 0.33 (− 0.74; 0.07)	− 0.29 (− 0.89; 0.3)	− 0.16 (− 0.56; 0.24)	− 0.03 (− 0.37; 0.32)	− 0.04 (− 0.16; 0.08)	0.01 (− 0.13; 0.15)	− 0.11 (− 0.32; 0.1)	− 0.12 (− 0.33; 0.08)	− 0.07 (− 0.19; 0.05)	− 0.05 (− 0.16; 0.06)
Δ(Cu to Zn ratio)	− 0.07 (− 0.48; 0.35)	0.32 (− 0.29; 0.93)	0.04 (− 0.37; 0.45)	− 0.06 (− 0.42; 0.29)	0.11 (− 0.01; 0.23)	− 0.02 (− 0.16; 0.13)	0.09 (− 0.12; 0.31)	0.08 (− 0.13; 0.3)	0.06 (− 0.06; 0.18)	0.02 (− 0.09; 0.13)
Δ(Se to Cu ratio)	− 0.19 (− 0.66; 0.27)	− 0.23 (− 0.9; 0.44)	− 0.14 (− 0.59; 0.31)	0.06 (− 0.32; 0.45)	− 0.07 (− 0.2; 0.07)	0.05 (− 0.11; 0.21)	− 0.07 (− 0.31; 0.17)	− 0.1 (− 0.34; 0.13)	− 0.08 (− 0.21; 0.05)	− 0.08 (− 0.2; 0.04)

ΔTE, defined as the differences between the follow-up concentration and the baseline concentration. Values are regression coefficients and corresponding 95% confidence intervals determined by multivariable linear regression analysis. Models are mutually adjusted for all the variables included in the Table

For comparability, dependent and independent variables (except categorical variables) are standardized

*Cu* Copper, *Fe* Iron, *I* Iodine, *Mn* Manganese, *Se* Selenium, *TE* Trace element, *Zn* Zinc

^a^Each parameter can be interpreted as change in the outcome, in SDs, per SD change in the predictors

Bold values denote significance

## Discussion

### Summary of the results with reference to study objectives

To our knowledge, the present study is the first characterizing the changing structure in TE profile during aging based on multiple TE (Mn, Fe, Cu, Zn, I and Se). In this German population of healthy elderly individuals, over a median follow-up period of 19 years, we observed a decrease in serum concentrations of Mn, Zn, Se, and the Se to Cu ratio and an increase in serum concentrations of Fe, Cu, I, and the Cu to Zn ratio. We were able to identify two factors of TE changes by PCA. The first factor reflected the correlated decrease in both Mn and Zn over time, whereas the second reflected the observed (on average) increase in both Cu and I.

### Comparison with other studies

#### Age-specific changes

Most studies investigating age-specific differences in TE status have been conducted cross-sectionally [13–15, 22, 23, 41–45] and comparison with the present work is, therefore, somewhat limited. However, overall, age-related changes in TE observed in our study were consistent with the literature. Two national surveys have examined predictors of blood Mn concentrations [23, 44]: one was conducted in a representative sample of Korean adults (KNHANES) and the other study used data from the US National Health and Nutrition Examination Survey. In the US survey [23], higher blood Mn concentration was associated with younger age, whereas the highest Mn concentrations were observed for individuals in the 30–39 age range in the Korean study [44]. In a Brazilian study [45] conducted among 947 adults, aged 40 year or older, blood concentrations of Mn were significantly lower with higher age. Possible comparison with our data is limited, as the above mentioned studies differ in the study design, in the population studied (e.g. younger or/and Asian individuals) and more importantly, in the assessment of Mn status. In these studies [23, 44, 45], Mn measurements were based on blood concentrations, and Mn whole-blood concentrations have been shown to be higher than serum Mn concentration as a considerable amount of Mn is bound to hemoglobin in erythrocytes [38]. In line with our findings, in the ZincAge study carried out among 1090 healthy elderly individuals aged of 74 years on average, negative correlations were observed between age and plasma Zn [22]. In that study based on data from five European countries, age was the most important predictor of Zn differences. According to the authors, this difference was due to physiological variations occurring during aging rather than dietary intake (assessed by dietary Zn intake and a Mediterranean diet score). Elevated plasma or serum Cu to Zn ratio has been suggested to represent a marker of health status and a predictor of all-cause mortality in elderly population [40]. Consistent with the literature [22, 40, 42], we observed that Cu to Zn ratio significantly increased with aging. In the present work, elevated Fe concentrations were observed with advancing age. These results contrast somehow with work indicating high prevalence of Fe deficiency anemia in older age [24], but appear in accordance with findings from the US Framingham Heart Study cohort which showed that white Americans aged 67–96 year old were at lower risk of Fe deficiency but had rather elevated Fe stores, based on multiple Fe measures [41]. However, it should be noted that serum Fe is not a good indicator of Fe stores and not a reliable tool for measuring Fe deficiency. Concerning Se status, our results are consistent with some previous studies, observing negative relationships between Se concentrations and age [13, 14, 43], while another study observed this association only in women [15]. In our study, median Se concentrations decreased from 85.19 (17.15) µg/L to 79.28 (17.69) µg/L after ~ 20y. This is consistent with the decline observed in the longitudinal EVA study of French elderly after 9 years of follow-up [46]. Se to Cu ratio has been suggested as a novel sensitive biomarker for resistance to thyroid hormone [31], as it allows to identify subjects with resistance to thyroid hormones due to mutations of the receptors. In our study, Se to Cu ratio significantly decreased over time, from 0.09 to 0.08. We observed herein a slight increase in serum concentrations of I during the period considered (1994–1996; 2004–2006). To our knowledge, no other study has evaluated age-specific differences in I using serum concentrations, limiting the comparability of the present results. Studies are rather based on other indicators such as urinary iodine concentration, or blood concentrations of thyroid stimulating hormone and thyroglobulin [47]. This trend is, therefore, rather difficult to interpret but might be seen in light of the fact that obligatory salt iodization has been stopped just after reunification in East Germany [48], and in turn the urinary I excretion has diminished at the beginning of the 1990s. This, however, needs to be considered with caution as improvement of I supply has been observed as of 1994 and variations existed across East Germany [48]. Of note, the recommendation for I intake does not vary for middle-age adults and older adults, notably because data are insufficient to support the need for deriving specific dietary reference values for older adults [49].

#### Interdependence between age-specific TE changes

Despite knowing that TE interact with each other and work in parallel, very few studies have looked at the interdependence between TE based on serum concentrations, limiting comparability of the present data with other studies. We investigated herein the correlations between differences in TE concentrations over time. In our study, low-to-mild correlations were observed between age-related differences in TE. The first factor identified by PCA reflected the correlated decline in both Mn and Zn over time while the second factor reflected the observed (on average) increase in both Cu and I over time. It should be noted that the two extracted-factors, based on only six variables, only explained 42% of the variance. Interestingly, increase in Cu concentrations over time were significantly positively correlated with increase in I concentrations. Although this needs further investigation, a possible hypothesis could be variation in food habits between the two time points. Thus, for example, seafood and fish are good sources of both I and Cu, and in turn the latter correlation may reflect changes in food intake over time. However, it cannot be determined whether the factors obtained reflect nutritional or/and metabolism change during aging. Further research is required to identify possible food determinants associated with specific TE patterns.

#### Determinants of TE changes

In the present work, we evaluated a large number of factors in relation to longitudinal TE variability, including factors related to change. Overall, the studied factors (mostly anthropometrics and lifestyle characteristics) were not strong determinants of TE longitudinal variability, except, predictably, due to the regression to the mean effect, the respective TE baseline concentration (higher TE concentration at baseline were related to higher decrease over time). Compared to men, we observed that women had lower increase in Cu and higher increase in Fe over time. Sex-specific differences in serum Cu concentrations have been repeatedly reported in earlier studies, with higher concentrations detected among women [42, 50]. Gender differences have also been observed for Fe. Interestingly, we also observed significant associations between change in vitamin and mineral use and longitudinal changes in Fe, Cu, I, and Se, suggesting that dietary supplement use may significantly affect overall TE status. To our knowledge, only one French study has investigated various factors in relation to longitudinal TE status, but limited to plasma Se [46]. In that study, in line with our results, sociodemographic and lifestyle characteristics had no effect on the slope of Se decline. However, hypertension had no effect on Se decline while surprisingly in our study we observed a negative relationship between Se decline and being hypertensive. The study observed an association of Se decline with occurrence of cardiovascular disease during follow-up. This finding cannot be confirmed by us as we excluded participants with cardiovascular disease from our study population. Herein, heavy alcohol consumers compared to low consumers had higher Fe increase during aging. This seems in line with studies showing that alcohol intake enhances Fe status. Higher dietary quality (as expressed by higher Mediterranean score) at baseline was related to higher Mn decrease. Further research investigating determinants of TE status should include nutritional factors, including food consumption and dietary supplement use as well as metabolic markers to disentangle their possible role and respective contribution to overall TE profiles.

#### Proportion of participants within normal TE concentrations

Inadequate supply of TE has been linked to several pathologic conditions [1]. In our study, participants were characterized by an overall good TE status, as assessed by the serum concentrations, except regarding Zn and Se, in particular at an advanced age. A low percentage of individuals in our study had concentrations outside reference ranges regarding Mn, in line with the fact that Mn deficiency is rare [1]. However, participants with Mn concentrations above the reference range were more frequent than those with low Mn concentrations (data not shown). In the present work, as highlighted above, a large number of individuals had their serum Fe concentrations within the reference ranges. Among the few individuals who had abnormal Fe concentrations in our study, rather high than low serum Fe concentrations were detected (data not shown), although not reflecting toxicity. This is noteworthy, as elevated Fe stores in middle-aged and older individuals have been linked to several chronic diseases [51]. However, as already mentioned, serum Fe concentration is less reliable to assess Fe status [51] than, e.g. plasma/serum ferritin [52]. Participants of our study had overall a good Cu status, as reflected by normal serum Cu concentrations. Serum Cu, which was used to assess Cu status in our study, is one of the most frequently used biomarkers for Cu status, despite its lack of specificity and limited sensitivity [52]. Based on nutritional intakes, the prevalence of Zn and Cu deficiency in healthy, middle-aged and older Europeans has been estimated to be < 11% for Zn and < 20% for Cu [21], contrasting somehow with our findings. In our study, a large percentage of the participants had Zn concentrations outside the reference ranges at both time points (almost half at baseline and 60% and after 20 years). It should be borne in mind that plasma Zn concentrations do not accurately reflect changes in Zn intake and status. However, despite its known weaknesses, circulating Zn remains widely used to determine Zn status. Given that serum Zn has limited reliability, developing more reliable biomarkers for Zn status is, therefore, still of public health relevance [1, 52], since mild Zn deficiency is very common [1]. The percentage of individuals with normal Se concentrations significantly decreased over time in our study. The optimal concentration for Se status, based on cut-off derived from saturation of selenoprotein P and glutathione peroxidase activity [39], was reached for only 13% of the participants at baseline and 6% at follow-up. These data may suggest insufficient intake of Se in this population of elderly German adults, in particular at advanced age. These figures are consistent, although slightly lower, with those observed in the SU.VI.M.AX study carried out cross-sectionally among middle-aged French individuals [15]. Increment of Cu to Zn ratio above 2 usually indicates inflammation or inadequate nutritional Zn status [40]. In our study, unsurprisingly, the percentage of individuals with Cu to Zn above this cutoff significantly increased with aging, reaching almost 20% at follow-up, indicating increased inflammatory response.

### Limitations and strengths

Main strengths of our study include its longitudinal design and its long follow-up period as well as the consideration of multiple essential TE to assess overall TE status and the use of validated methods for the measurement of the different TE. Some limitations of our study should be noted. First, serum TE are not always the most sensitive markers of TE status (e.g. Zn or Cu). Research for suitable and specific biomarkers of TE status is still relevant, while considering factors such as health condition, sex or age. Another limitation is also that only one sample of each TE was measured at each time point. In addition, the sample size was relatively small which may have limited the statistical power. However, even though the sample size was small, we were able to observe significant changes over time. Furthermore, our study concentrated on healthy elderly participants (free or major chronic diseases for the whole study period and not users of antibiotics, metformin, and statins at the two time points). While disease information was collected during the whole follow-up, information on medication was only available at the two specific time points, which may have restricted the analysis findings, as the use of certain medication prior examination may have affected the TE status of the participants. Potential degradation over time is also possible. Although it cannot totally be ruled out, it is unlikely that has affected our findings. Total element concentrations were measured and since the species does not matter in ICP-MS/MS, potential degradation may be related to volatilisation which is very unlikely as the samples were frozen at − 80 °C in closed containers, and the elements of interest do not form any volatile species under these conditions. Another possible degradation could be leaching from the storage containers. However, the containers used are very unlikely to contain our analytes in amounts that would significantly change the outcome of our study. As some data were missing, we also imputed some variables. In the sensitivity analyses restricted to individuals with available data, the trends concerning the TE changes were comparable to those from our imputed analyses.

## Conclusions

In conclusion, in this population-based study of elderly individuals, decrease in Mn, Zn, and Se concentrations and increase in Fe, Cu, and I concentrations were observed over 20 years of follow-up. Further research is required to identify possible food and dietary determinants as well as biomarkers associated with specific TE patterns, along with disease-specific TE profiles.

## Electronic supplementary material

Below is the link to the electronic supplementary material.

Supplementary material 1 (DOCX 31 kb)

## Abbreviations

Cu: Copper
Fe: Iron
I: Iodine
Mn: Manganese
PCA: Principal component analysis
Se: Selenium
TE: Trace element
Zn: Zinc
