# Supplementary material for: Changes of trace element status during aging: results of the EPIC-Potsdam cohort study
Source: Eur J Nutr. 2019 Nov 30;59(7):3045–58. doi: 10.1007/s00394-019-02143-w (PMC7501115; doi:10.1007/s00394-019-02143-w)
Supplement: Supplementary file 1 — Supplementary material 1 (DOCX 31 kb) [file 394_2019_2143_MOESM1_ESM.docx]

Electronic Supplementary Material

**Changes of trace element status during aging: results of the EPIC-Potsdam cohort study**

Julia Baudry^1, 2^, Johannes F. Kopp^2, 3^, Heiner Boeing^4^, Anna P. Kipp^2, 5^, Tanja Schwerdtle^2, 3^, Matthias B. Schulze^1, 2^

^1^ Department of Molecular Epidemiology, German Institute of Human Nutrition, Potsdam-Rehbrücke, 14558 Nuthetal, Germany

^2^ TraceAge – DFG Research Unit on Interactions of Essential Trace Elements in Healthy and Diseased Elderly, Potsdam-Berlin-Jena, Germany

^3^ University of Potsdam, Institute of Nutritional Science, Department of Food Chemistry, 14558 Nuthetal, Germany

^4^ Department of Molecular Epidemiology, German Institute of Human Nutrition, Potsdam-Rehbrücke, 14558 Nuthetal, Germany

^5^ Friedrich Schiller University Jena Institute of Nutritional Sciences Department of Molecular Nutritional Physiology, 07743 Jena, Germany

**Corresponding author:** [julia_aida.baudry@dife.de](mailto:julia_aida.baudry@dife.de)

**Supplemental Table 1:** ICP-MS/MS instrument parameters

| **ICP-MS/MS parameters** | |  |  | |
| --- | --- | --- | --- | --- |
| **instrument** | Agilent 8800 ICP-QQQ-MS, equipped with integrated autosampler | | | |
| **plasma RF power** | 1550 W |  | | |
| **nebulizer** | Glass Expansion MicroMist | | |  |
| **spray chamber** | Scott-type |  | | |
| **plasma gas flow** | 15.0 L/min |  | | |
| **nebulizer gas flow** | 1.20 L/min |  | | |
| **spray chamber temperature** | 2°C |  | | |
| **cones** | Ni |  | | |
| **sample depth** | 8.0 mm |  | | |
| **gas modes** | **He** | **O_2_** | | |
| **cell gas flow**  **stabilization time** | 3.0 mL/min He  18 s | 0.6 mL/min O_2_  18 s | | |
| **m/z Q1** | 55 (Mn), 56 (Fe), 63 (Cu), 66 (Zn), 103 (Rh), 127 (I) | 77 (Se), 80 (Se), 103 (Rh) | | |
| **m/z Q2** | 55 (Mn), 56 (Fe), 63 (Cu), 66 (Zn), 103 (Rh), 127 (I) | 93 (Se), 96 (Se), 103 (Rh) | | |
| **integration time** | 0.5 s | 0.5 s | | |
| **replicates** | 3 | 3 | | |

Abbreviation: ICP-MS/MS, Inductively coupled plasma tandem mass spectrometry.

**Supplemental Table 2**: Serum concentrations of measured TE at baseline and at 20y of follow-up, EPIC-Potsdam, N=203^a^

|  | At baseline  (1994-1998) | At 20y follow-up  (2014-2016) | P-value^b^ | Absolute difference^c^ | Relative difference^d^ (%) |
| --- | --- | --- | --- | --- | --- |
| **Manganese (µg/L)** | 1.06 (1.11) | 0.83 (0.88) | 0.0019 | 0.14 (1.13) | -15 (109) |
| **Iron (µg/L)** | 955 (446) | 1114 (376) | <.0001 | -179 (459) | 19 (55) |
| **Copper (µg/L)** | 996 (227) | 1042 (236) | <.0001 | -54 (186) | 5 (20) |
| **Zinc (µg/L)** | 702 (173) | 634 (149) | <.0001 | 48 (193) | -7 (26) |
| **Iodine (µg/L)** | 47.85 (12.26) | 49.26 (10.48) | 0.002 | -1.99 (9.40) | 5 (20) |
| **Selenium (µg/L)** | 84.54 (16.67) | 79.11 (17.14) | <.0001 | 5.61 (16.09) | -7 (18) |
| **Cu to Zn ratio** | 1.43 (0.48) | 1.62 (0.52) | 0.002 | -0.20 (0.49) | 14 (36) |
| **Se to Cu ratio** | 0.09 (0.02) | 0.08 (0.02) | 0.002 | 0.01 (0.02) | -10 (24) |

^a^ Data are medians (IQR).

^b^ P-values based on non-parametric Wilcoxon signed-rank test.

^c^ Differences between the baseline concentration and the follow-up concentration.

^d^ Differences between the follow-up concentration and the baseline concentration, with respect to the baseline concentration, multiplied by 100.

Abbreviation: Cu to Zn ratio, Copper to Zinc ratio; Se to Cu ratio, Selenium to Copper ratio; TE, Trace element.

**Supplemental Table 3**: Percentage of individuals within the reference ranges at baseline and at 20y of follow-up, EPIC-Potsdam, N=203

|  | Normal reference range [µ/L] | % at baseline | % at 20 y follow-up | Crude P-value^a^ | Adjusted P-value^b^ |
| --- | --- | --- | --- | --- | --- |
| **Manganese** | 0.15–2.65 | 86.7 | 94.09 | 0.0071 | 0.0189 |
| **Iron** | Men: 550-1600;  Women: 400-1550 | 92.12 | 92.61 | 0.8575 | 0.8575 |
| **Copper** | 637-1401.2 | 92.12 | 95.07 | 0.1336 | 0.1527 |
| **Zinc** | 660-1100 | 53.2 | 41.38 | 0.0114 | 0.0228 |
| **Iodine** | 40-92 | 84.24 | 88.67 | 0.1282 | 0.1527 |
| **Selenium** | 70-150 | 89.66 | 75.86 | 0.0001 | 0.0008 |
| Optimal status | >100 | 12.32 | 6.4 | 0.0339 | 0.0542 |
| **Cu to Zn ratio** | ≤2 | 91.13 | 82.76 | 0.0041 | 0.0164 |

^a^ P-values based on McNemar’s test.

^b^ False Discovery Rate adjusted p-values.

Abbreviation: Cu to Zn ratio, Copper to Zinc ratio.

**Supplemental Table 4:** Spearman correlations between TE differences over time, EPIC-Potsdam, N=203^a^

|  |  | Δ**Mn** | Δ**Fe** | Δ**Cu** | Δ**Zn** | Δ**I** | Δ**Se** |
| --- | --- | --- | --- | --- | --- | --- | --- |
| Δ**Mn** | r | 1 |  |  |  |  |  |
|  | P | . |  |  |  |  |  |
| Δ**Fe** | r | 0.03 | 1 |  |  |  |  |
|  | P | 0.63 | . |  |  |  |  |
| Δ**Cu** | r | **0.10** | -0.04 | 1 |  |  |  |
|  | P | 0.15 | 0.59 | . |  |  |  |
| Δ**Zn** | r | **0.14** | **0.14** | **0.16** | 1 |  |  |
|  | P | 0.04 | 0.05 | 0.03 | . |  |  |
| Δ**I** | r | -0.03 | 0.01 | **0.30** | **0.17** | 1 |  |
|  | P | 0.63 | 0.89 | <0001 | 0.02 | . |  |
| Δ**Se** | r | -0.01 | -0.04 | 0.01 | **0.14** | 0.07 | 1 |
|  | P | 0.89 | 0.54 | 0.93 | 0.05 | 0.31 | . |

^a^ ΔTE, defined as the differences between the follow-up concentration and the baseline concentration. Correlation coefficients r ≥ 0.10 or r ≤ -0.10 are displayed in bold.

Abbreviation: Cu, Copper; Fe; Iron; I, Iodine; Mn, Manganese; P, significance or probability value; r, correlation coefficient; Se, Selenium; TE, Trace element; Zn, Zinc.

**Supplemental Table 5**: Spearman partial correlations between TE differences over time, adjusted for other TE differences, EPIC-Potsdam, imputed sample, N=219^a^

|  |  | **ΔMn** | **ΔFe** | **ΔCu** | **ΔZn** | **ΔI** | **ΔSe** |
| --- | --- | --- | --- | --- | --- | --- | --- |
| **ΔMn** | r | 1 |  |  |  |  |  |
|  | P | . |  |  |  |  |  |
| **ΔFe** | r | 0.01 | 1 |  |  |  |  |
|  | P | 0.91 | . |  |  |  |  |
| **ΔCu** | r | **0.10** | -0.02 | 1 |  |  |  |
|  | P | 0.16 | 0.74 | . |  |  |  |
| **ΔZn** | r | **0.11** | **0.12** | 0.05 | 1 |  |  |
|  | P | 0.09 | 0.07 | 0.49 | . |  |  |
| **ΔI** | r | -0.06 | -0.01 | **0.29** | **0.18** | 1 |  |
|  | P | 0.35 | 0.89 | <.0001 | 0.01 | . |  |
| **ΔSe** | r | -0.01 | -0.01 | 0.02 | **0.11** | 0.04 | 1 |
|  | P | 0.87 | 0.88 | 0.83 | 0.11 | 0.53 | . |

^a^ ΔTE, defined as the differences between the follow-up concentration and the baseline concentration.

Correlation coefficients r ≥ 0.10 or r ≤ -0.10 are displayed in bold.

Abbreviation: Cu, Copper; Fe; Iron; I, Iodine; Mn, Manganese; P, significance or probability value; r, correlation coefficient; Se, Selenium; TE, Trace element; Zn, Zinc.

**Supplemental Fig. 1:** Factor loadings after varimax rotation of the two PCA-derived factors based on TE concentration differences over time, EPIC-Potsdam, N=203

ΔTE, defined as the differences between the follow-up concentration and the baseline concentration.

Abbreviation: Cu, Copper; Fe; Iron; Mn, Manganese; I, Iodine; PCA, Principal component analysis; Se, Selenium; TE, Trace element; Zn, Zinc.
